# Identification and functional characterisation of the promoter of the calcium sensor gene *CBL1 *from the xerophyte *Ammopiptanthus mongolicus*

**DOI:** 10.1186/1471-2229-10-18

**Published:** 2010-01-29

**Authors:** Lili Guo, Yanhua Yu, Xinli Xia, Weilun Yin

**Affiliations:** 1 National Engineering Laboratory for Tree Breeding, College of Biological Sciences and Biotechnology, Beijing Forestry University, No. 35, Tsinghua East Road, Beijing, PR China

## Abstract

**Background:**

*CBL1 *is a calcium sensor that regulates drought, cold and salt signals in *Arabidopsis*. Overexpression of *CBL1 *gene in *Arabidopsis *and in *Ammopiptanthus mongolicus *showed different tolerant activities. We are interested in understanding the molecular mechanism of the upstream region of the *CBL1 *gene of *A. mongolicus *(*AmCBL1*). We investigated and characterized the promoter of the *AmCBL1 *gene, for promoters play a very important role in regulating gene expression in eukaryotes.

**Results:**

A 1683-bp 5' flanking region was isolated from *A. mongolicus*. The sequence was identified as *AmCBL1 *promoter. Analysis of the promoter sequence indicated a 690-bp intron and some basic cis-acting elements were related to various environmental stresses and plant hormones. To identify the functional region of the *AmCBL1 *promoter, five plant expression vectors fused with the GUS (β-glucuronidase) gene, driven by series deleted fragments of *AmCBL1 *promoter at different lengths from -1659, -1414, -1048, -296 to -167 bp relative to the transcriptional start site were constructed and transformed into *Nicotiana tabacum *L. cv. 89. Functional properties of each promoter segment were examined by GUS staining and fluorescence quantitative analyses using at least three single-copy PCR-positive plants of transgenic tobacco, treated with various environmental stresses and plant hormones for different times. We demonstrated that the *AmCBL1 *promoter was a vascular-specific and multiple-stress-inducible promoter. Our results further imply that the promoter fragment B1S3 possessed sufficient essential cis-acting elements, accounting for vascular-specific and stress-induced expression patterns. It may also indicate that for response to some stresses certain cis-elements are required in tissues outside the region of the B1S3 construct.

**Conclusions:**

To help resolve uncertainties about the upstream regulatory mechanism of the *CBL1 *gene in desert plants, we suggest that the function of the *AmCBL1 *promoter, particularly under conditions of abiotic stress, to be examined for possible usefulness in molecular breeding. Regardless of the outcome, the allocation and relative quantification of the GUS-fusion *AmCBL1 *promoter segments at transcriptional levels in different tissues under various stresses across separate promoter segments suggests that the *AmCBL1 *promoter is a phloem-specific and multiple-stress-inducible promoter. These data coupled with the ongoing *AmCBL1 *5' UTR intron analyses provide a solid foundation for their future use in molecular breeding as new promoters of stress-resistance genes from desert plants.

## Background

Desert ecosystems currently cover about 35% of the Earth's land surface [1]. Additionally, water deficiency has become a worldwide problem. This desertification in arid and semi-arid regions, as well as water deficiency, have been become a focus of attention internationally [2]. It is urgent that stress-related genes and their upstream regulatory mechanism, together with stress-resistant species be studied extensively.

Calcium is known for its crucial role as a second messenger in mediating multiple defence responses under various environmental stress stimuli [3-6]. Primary calcium sensor Calcineurin B-like protein (*CBL*) which was identified as a calcium binding protein was recently isolated in higher plants [7-9]. Recent studies indicate that the *CBL *protein is not only an integrative node responding to stress stimulus, but also an upstream regulator of stress gene expression in plants [10-12]. Overexpression of *CBL *could confer several stress tolerance [13,14].

In the *CBL *gene family, *CBL1 *can be induced by various stress signals, such as wounding, cold, drought and high salinity [10]. Interestingly, overexpression of this gene in *Arabidopsis *showed enhanced tolerance to drought and salt, but reduced tolerance to freezing [15-17]. We observed that overexpression of the *CBL1 *gene enhanced tolerance to both drought and cold (data not shown) in *A. mongolicus*. We predicted that this kind of expression difference might be caused by the upstream regulatory effect.

Although the *CBL1 *gene is an important point in the calcium signal transduction pathway, little is known about its regulatory mechanism. Moreover, recent studies on *CBL1 *have focused on *Arabidopsis*, instead of on other species with stronger stress tolerance. *A. mongolicus *is the only super-xerophytic evergreen broadleaf shrub species growing in the desert region of northwest China. It has extremely strong tolerance to drought, cold, heat, solar radiation stress and poor site qualities [18], and can survive a winter temperatures less than -30°C [19]. Up to now, studies on *A. mongolicus *have mainly focused on antifreeze proteins, genetic diversity, spatial heterogeneity of soil and the cold-induced *AmCIP *gene [20-23]. Dissecting the upstream regulatory part of the *A. mongolicus CBL1 *gene should contribute to understanding the molecular mechanisms of calcium signal cascades in a calcium sensor relay. A stress-inducible promoter of *CBL1 *is also a good resource for transgenic engineering.

In this study, the1683-bp 5'-flanking region of *AmCBL1 *promoter was isolated and analysed, and an intron was found in the 5'-UTR region. We also investigated the role of this promoter region, with regard to its tissue-specific expression pattern and relative expression activities, using transgenic analyses in tobacco (which has a same expression pattern as *A. mongolicus*). In addition, we demonstrated the shortest promoter region sufficient for tissue-specific expression and the stress-induced expression activity.

## Results

### Isolation of *AmCBL1 *promoter

**Figure 1:**
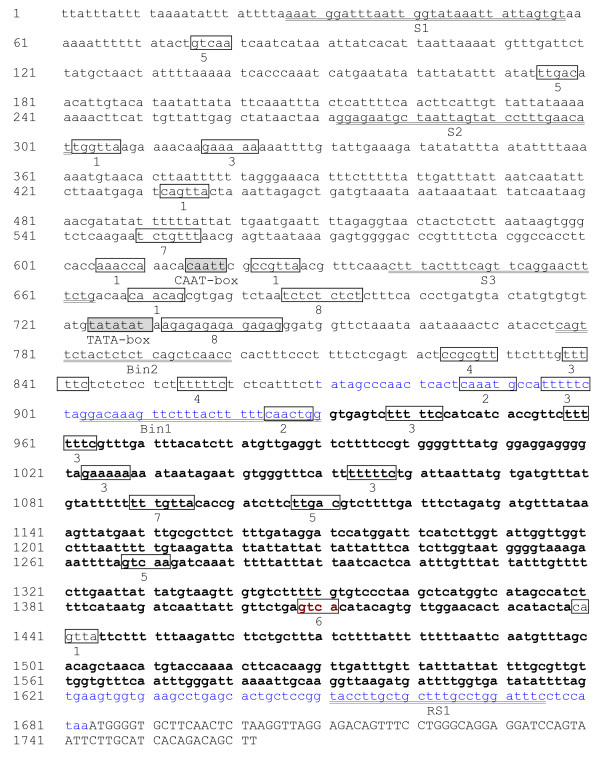
**Promoter sequence of *AmCBL1***. Capital character: part coding region of *AmCBL1*; Blue sequence: 5'UTR; Bold sequence: the intron was 5'-upstream of *AmCBL1*; Underlined sequence: primers designed for deletion analysis; 1: MYB recognition site; 2: MYC recognition site; 3: GT1GMSCAM4: pathogen- and salt-responsive element; 4: ABRERATCAL: Ca-responsive element; 5: WBOXATNPR1: SA-responsive element; 6: WBOXHVISO1: sugar-responsive element; 7: GAREAT: GA-responsive element; 8: CTRMCAMV35S: enhancer.

We isolated the *AmCBL1 *promoter with the detailed amplification procedure shown in Table 1. Since the sequence of the first exon of *AmCBL1 *gene was only 79 bp in length, it was difficult to design three gene-specific primers based on it. For extending the primer designing space, we cloned the first intron of the *AmCBL1 *gene using primers INT1/INT2 designed on the first and second exon of the *AmCBL1 *gene. Then chromosome walking was employed for promoter cloning. Then one 850-bp sequence was obtained after the longest strip was sequenced. Using the same method, primers GSP4, GSP5, GSP6, GSP7, GSP8, and GSP9 were designed to walk further on the basis of the newly sequenced fragment, and sequences of 456 and 374 bp were obtained separately. A 1683-bp 5' flanking region was finally obtained (Figure. 1). A putative 'TATA' box motif 'TATATATA' was found 150 bp upstream of the initiation codon ATG of the *AmCBL1 *open reading frame. The sequence upstream of the transcription start, located at 872 (blue label), is the whole promoter region All primer sequences are in Table 2.

**Table 1:** Amplification procedure of *AmCBL1 *promoter

Step	procedure	primers	template
First intron amplification		INT1/INT2	genome DNA

First walking	linear amplification reaction add poly C to end	GSP1	genome DNA
	amplification of poly-added production	GSP2/AAP	poly-added production
	amplification of target gene	GSP3/AUAP	last step production

PCR amplification		W1/GSP2	genome DNA

second walking	linear amplification reaction add poly C to end	GSP4	genome DNA
	amplification of poly-added production	GSP5/AAP	poly-added production
	amplification of target gene	GSP6/AUAP	last step production

PCR amplification		W1/GSP2	genome DNA

third walking	linear amplification reaction add poly C to end	GSP7	genome DNA
	amplification of poly-added production	GSP8/AAP	poly-added production
	amplification of target gene	GSP9/AUAP	last step production

*AmCBL1 *promoter amplification		S1/GSP2	genome DNA

**Table 2:** Primers used in this experiment

Primer	sequence
INT1	GGTGCTTCAACTCTAAGG
INT2	CAAATAGTGCTTCAACCTC
GSP1	CAGATTTGCATCATTCCATTGAGAAGAC
GSP2	GTCTGTGATGCAAGAATTACTG
GSP3	GTCTCCTAACCTTAGAGTTGAAGCAC
GSP4	TTCTACCCCTCCTCCCATAAAC
GSP5	CCACGGAAAAGAACCTCCACATAAG
GSP6	CGGTGATGATGGAAAAAGACTC
GSP7	CATCAGCTCTAATTTAGTAACTGATCTCATT
GSP8	CTTAACCAATGTTCAAAGGATACTAATTAGCATTC
GSP9	TCCTTTAGTTATAGCTCAATAACAATGAAG
GSP0	GTGCTTCAACCTCGCTGACAG
AAP	GGCCACGCGTCGACTAGTACGGGIIGGGIIGGGIIG
AUAP	GGCCACGCGTCGACTAGTAC
S1	AAATGGATTTAATTGGTATAAATTATTAGTGT
S2	GGAGAATGCTAATTAGTATCCTTTGAACAT
S3	CTTTACTTTCAGTTCAGGAACTTTCTG
HS1	CTAAGCTTAAATGGATTTAATTGGTATAAATTATTAGTGT
HS2	CTAAGCTGGAGAATGCTAATTAGTATCCTTTGAACAT
HS3	CTAAGCTTCTTTACTTTCAGTTCAGGAACTTTCTG
RS	ATGGAGGAAATCCAGGCAAAG
Bin1	CCAGTTGAAAAAGTAAAGAACTTTGTCC
Bin2	GGTTGAGCTGAGAGAGTAGAACTG
Ins	CTAAGCTTTACTTTTTCAACTGGGTGAGTCTT
Inr	CTAAGCTTTGCTCAGGCTTCACCACTTC

### Multiple environmental-related and hormone-related cis-elements were predicted on *AmCBL1 *promoter sequence

Using PLACE and PLANTCARE databases, we analysed the sequence of *AmCBL1 *and *AtCBL1 *promoters, and predicted their key cis-acting elements and the location of these elements. Thus, *AmCBL1 *promoter was shown to harbour multiple stress cis-acting elements (Table 3). Eight homologue sequences of the pathogenesis- and salt-related cis-acting element GT1GMSCAM4 were found there. One homologue sequence of CPBCSPOR, which is a specific binding site of cytokinin-dependent protein [24], was discovered. Two homologue sequences of GAREAT, which is abundant upstream of GA-induced genes in *Arabidopsis *[25], were examined. Three homologue sequences of WBOXATNPR1 were also detected. Two homologue sequences of WBOXHVISO1 which mainly participates in the sugar signal transduction [26] were found. In addition, light-responsive elements were found, such as I-BOX, GT1 and GATABOX. Only one homologue sequence of ABRERATCAL element was discovered. ABRERATCAL is a calcium responsive cis-element that exists in 162 unregulated genes [27]. We also found that two CT/GA-rich motifs existed near the TATA-box of the *AmCBL1 *promoter.

**Table 3:** Prediction cis-elements of *AmCBL1 *promoter, *AtCBL1 *promoter and *AtRD29A *with database analysis

Element	Element core sequence	Element number			Function
		*AmCBL1P*	*AtCBL1P*	*AtRD29A*	
ABRERATCAL	MACGYGB	1	3	1	response to calcium ion
CGCGBOXAT	VCGCGB	2	2	2	involved in multiple signal transduction
CTRMCAMV35S	TCTCTCTCT	2	1	0	plays as enhancer
CPBCSPOR	TATTAG	1	4	2	response to CTK signal
CURECORECR	GTAC	10	8	2	copper and oxygen signals
GATABOX	GATA	8	14	6	response to light signal
GT1CONSENSUS	GRWAAW	18	21	14	response to light signal
IBOXCORE	GATAA	3	5	3	response to light signal
INRNTPSADB	YTCANTYY	7	5	4	response to light signal
GT1GMSCAM4	GAAAAA	9	9	4	pathogenesis and salt-related
MYB	CNGTTR/WAACCA/YAACKG	6	10	5	response to drought and ABA signals
MYC	CANNTG/CATGTG/CACATG	2	3	6	response to drought, ABA and cold signals
NTBBF1ARROB	ACTTTA	2	2	0	response to auxin signal
TAAAGSTKST1	TAAAG	8	3	2	regulate guard cell-specific gene expression
GAREAT	TAACAAR	2	1	2	response to GA signal
WBOXNTERF3	TGACY	3	3	3	response to wound signal
WBOXATNPR1	TTGAC	3	4	3	response to SA signal
WBOXHVISO1	TGAC	2	3	2	response to sugar signal

Since a stress-inducible promoter in other xerophytic plants has never been reported in spite of extensive research in *Arabidopsis*, we compared the *AmCBL1 *promoter with the *AtCBL1 *and *AtRD29A *promoters, respectively, using database analysis. The result showed that the cis-acting elements predicted were nearly identical with each other, despite their low homology (Table 3). However, the number of cis-elements predicted differed. The above database analysis showed that most elements existing in *AmCBL1 *promoter were mainly environment- or hormone-responsive motifs. We therefore predicted that *AmCBL1 *promoter would be an inducible promoter and regulated by multiple abiotic factors and hormones. We conjectured that expression of the *AmCBL1 *gene under normal conditions might be higher than of the *AtCBL1 *gene, possibly due to the adaptation mechanisms of this xerophytic plant to abiotic stresses. It is interesting that a 690-bp intron was found on 5'-UTR of the *AmCBL1 *promoter, 63 bp away from the initiator ATG of the *AmCBL1 *gene, by BLASTing sequences of *AmCBL1 *promoter and the untranslated 5'-region (5' UTR) of *AmCBL1 *cDNA (data not published) (see Additional file 1 and Additional file 2).

### All the five promoter deletion segments were sufficient to drive GUS expression in transient expression

**Figure 2:**
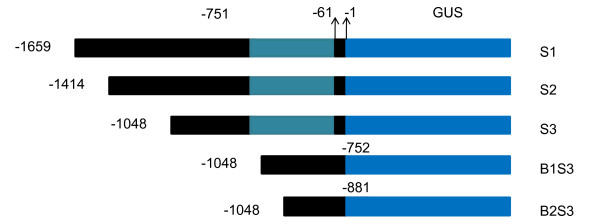
***AmCBL1 *promoter deletion analysis**. A in translation start site ATG represents +1; the first base before ATG represents -1; S1: full-length of *AmCBL1 *promoter; S2: S1 5'-end deletion of 245 bp; S3: S2 5'-end deletion of 366 bp; B1S3: 3'-end deletion of intron based on S3; B2S3: 3'-end deletion of 129 bp based on B1S3.

To study the contribution of different regions of the *AmCBL1 *promoter to its expression activity, we first performed a comparative transgenic analysis of five different promoter-GUS fusion constructs (Figure. 2). All of them were separately transferred into tobacco (*Nicotiana tabacum *L.) cv. 89 and *A. mongolicus *by *Agrobacterium*-mediated gene transfer. Our result revealed all the five promoter deletion segments were sufficient to drive GUS expression by transient expression both in *A. mongolicus *and tobacco (Additional file 3). A discrepancy of GUS expression pattern between *A. mongolicus *and tobacco lied with the exact expression locus. GUS staining was detected in the main leaf veins in tobacco, whereas blue points were observed only in leaf margins of *A. mongolicus*. This might have resulted from the different leaf properties of the two plants. Tobacco leaves are very thin, making gene transfer easier, while *A. mongolicus *has fleshy leaves that could prevent transformation.

### Regeneration of transgenic tobacco plants

To further identify the functional regions of the *AmCBL1 *promoter, a series of chimeric GUS genes containing 5'-deletion derivatives of the 1683-bp *AmCBL1 *promoter (Figure. 2) were separately transferred intotobaccocv.89 by *Agrobacterium*-mediated leaf-disc transformation. Transgenic tobacco lines were obtained, belonging to six groups (S1, S2, S3, B1S3, B2S3 and CaMV35S). Three independent kanamycin-resistant PCR-positive (see Additional file 4) single-copy (see Additional file 5) transgenic tobacco plants from each of group were chosen for further analysis. The detailed experimental procedure is described in Materials and Methods.

### Histochemical analysis of GUS activities in transgenic tobacco seedlings

**Figure 3:**
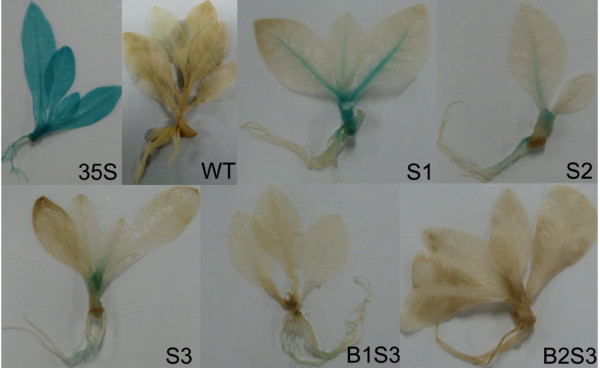
**Histochemical analysis of GUS activity**. GUS expression activity was detected by vacuuming seedlings in X-Gluc solution overnight using one-week-old transgenic tobacco seedlings, directed by *AmCBL1 *promoter construct S1, S2, S3 and B1S3. For positive and negative controls, 35S and WT were used separately.

To identify the expression profiles of transgenic plants driven by the five *AmCBL1 *promoter deletion segments and the CAMV35S promoter, the independent transgenic plants were subjected to histochemical stains as described in Materials and Methods. The expression profiles of all five transgenic *AmCBL1 *promoter constructs are shown in Figure. 3. GUS expression was detected in transgenic seedlings of S1, S2, S3, B1S3, B2S3, 35S and wild type tobaccos using one-week-old etiolated seedlings. The CaMV35S promoter was expressed in all tobacco tissues, and no GUS activity was detected in wild-type tobacco; however, GUS staining was detected in leaf veins, stems and roots of plants containing the full-length promoter construct S1, and deletion promoter constructs S2, S3 and B1S3. No GUS expression was detected in the longest deletion construct B2S3. In addition, GUS expression activity for S1 was the strongest among the five *AmCBL1 *promoter constructs examined; S2 and S3 had similar expression patterns to each other. Only detectable blue points were examined in B1S3, and deletion of the 129 bp from the region of B1S3 resulted in no GUS activity in transgenic plants. This implies that the *AmCBL1 *promoter was tissue-specific and that the promoter deletion segment B1S3 was sufficient to drive gene expression, and also that the 129-bp deleted-fragment might contain some cis-acting elements.

### GUS expression localisation of *AmCBL1 *promoter segments

**Figure 4:**
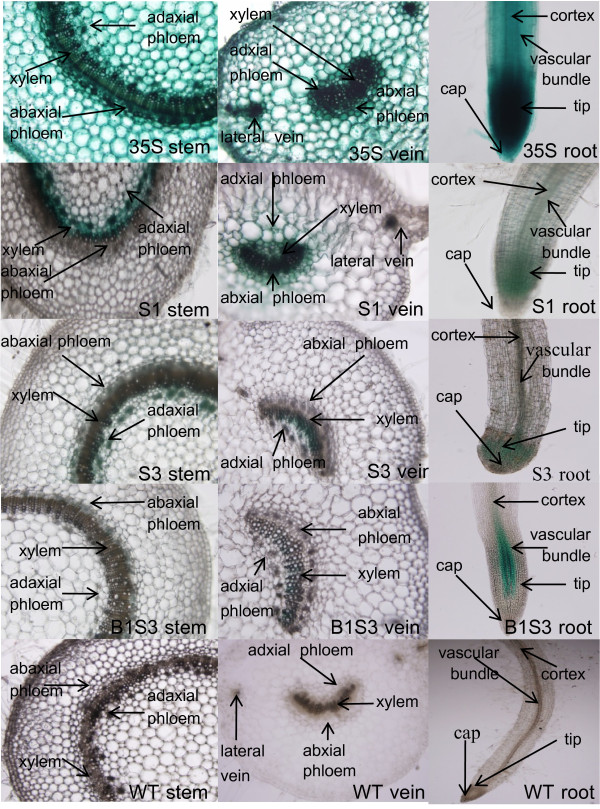
**Histochemical localisation of GUS activity**. Razorblade sections in roots, petioles and stems of transgenic tobacco plants transformed with constructs S1, S3, B1S3, 35S and WT. Transverse petiole and stem sections and from S1, S2, S3, 35S and WT were stained with X-Gluc in NaH_2 _P04, pH 7.0, overnight at 37°C. Vertical root sections from the above constructs were also stained overnight with X-Gluc as above. Positive and negative controls were 35S and WT, used separately.

A GUS staining experiment by razorblade section was used to further investigate the exact location of GUS gene expression. Since the constructs S2 and S3 had similar expression activity, and construct B2S3 had no GUS activity, we chose S1, S3 and B1S3 for further experiments. Transgenic plants driven by the CaMV35S promoter, and wild-type tobacco, were the respective positive and negative controls. Blue points were detected in vascular bundles, mesophyll and cortex of leaves; vascular bundles, pith and cortex of stems; and vascular bundles, tips and caps of roots of transgenic tobacco driven by the CaMV35S promoter (Figure. 4). No GUS staining was observed in tissues of wild-type tobacco. For transgenic tobacco driven by promoter segments S1, S3 and B1S3, there was GUS staining only in vascular bundles, especially phloem of leaf veins, stems, and roots. It is interesting that GUS expression was only detected in the meristematic zone of root tips, and no blue staining was observed in root caps (Table 4). We conjecture that the *AmCBL1 *promoter was a vascular-specific and particularly a phloem-specific promoter. Moreover, the full-length promoter construct S1 still showed the strongest GUS expression activity of the three *AmCBL1 *promoter constructs examined. Since there was a sharp decrease in GUS staining in the distal segment B1S3, when the intron was deleted from S3 3'-end, we suggest that the promoter expression strength was mainly determined by this 5'-UTR intron. This also suggests that this 5'-UTR intron might contain certain enhancer-like elements necessary for promoter activity.

**Table 4:** GUS protein localization of transgenic tobaccos directed by construct S1, S3 B1S3

segment	leaf			stem			root		
	
	Vascular bundle	mesophyll	cortex	Vascular bundle	pith	cortex	Vascular bundle	tip	cap
S1	+	-	-	+	-	-	+	+	-
S3	+	-	-	+	-	-	+	+	-
B1S3	+_	-	-	+	-	-	+	+	-
35S	+	+	+	+	+	+	+	+	+
CK	-	-	-	-	-	-	-	-	-

### Fluorometric analysis of GUS activities in transgenic tobacco

**Figure 5:**
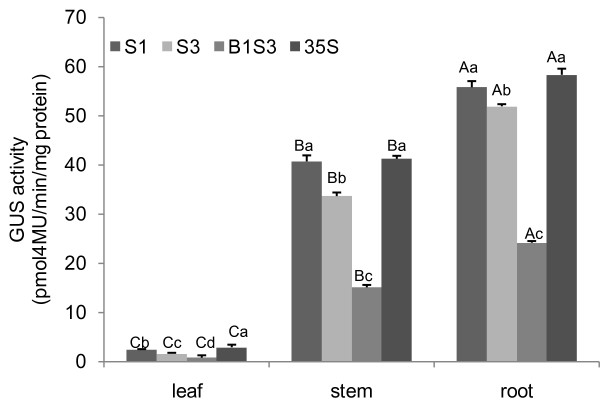
**Fluorometric quantification of GUS activity**. GUS enzyme activity among different transgenic groups in leaves, stems and roots. The GUS activity is expressed in μg 4-methylumblliferone min^-1 ^mg^-1 ^protein, and a graph drawn of the average rate of GUS activity per collection of transgenics per construct. The quantification of GUS activity for each promoter construct was replicated three times. Statistical analysis was performed using least significant difference and homogeneity of variance test by SPSS 16.0, and one way ANOVA test was used for the statically analysis. Means with different lower-case or upper-case letters were statistically different at *P *< 0.05 among segments and between tissues, respectively. Error bars on the graph represent SE with three replicates.

The expression quantity of different *AmCBL1 *promoter segments in different transgenic tobacco tissues (leaf, stem and root) under normal conditions are shown in Figure. 5. The GUS-specific signal was very high in roots and stems, while leaf samples showed only visible GUS activities. There was a gradual decrease in GUS activities upon deletion of the *AmCBL1 *promoter. The full-length promoter segment S1 had the highest GUS activity of the three promoter constructs; however, it was still lower than the positive-construct CaMV35S promoter in roots, stems or leaves. Results of the GUS assay on intron-less promoter segment B1S3 showed that removing the 690-bp intron significantly affected quantitative behaviour; GUS activity decreased sharply with intron deletion.

There were significant differences among leaf, stem, and root. There were also significant differences among S1, S3, B1S3 and 35S in leaves. For stems and roots, there were significance differences in S1, S3 and B1S3 (Figure. 5). Significant differences among S1, S3 and B1S3 in leaves, stems or roots all support the above result that the 611-bp deletion segment and the 690-bp deletion 5'-UTR intron might contain an enhancer-like cis-element. The fluorescence quantification data correlated with the histochemical staining results. Together with the GUS staining results, we demonstrated that the 611-bp deletion sequence and the 5'-UTR intron were required for both tissue specificity and quantitative behaviour. Moreover, the 5'-UTR might play a role as an enhancer-like expression module.

### Effects of stresses on gene expression of *AmCBL1 *promoter segments in tobacco seedlings

**Figure 6:**
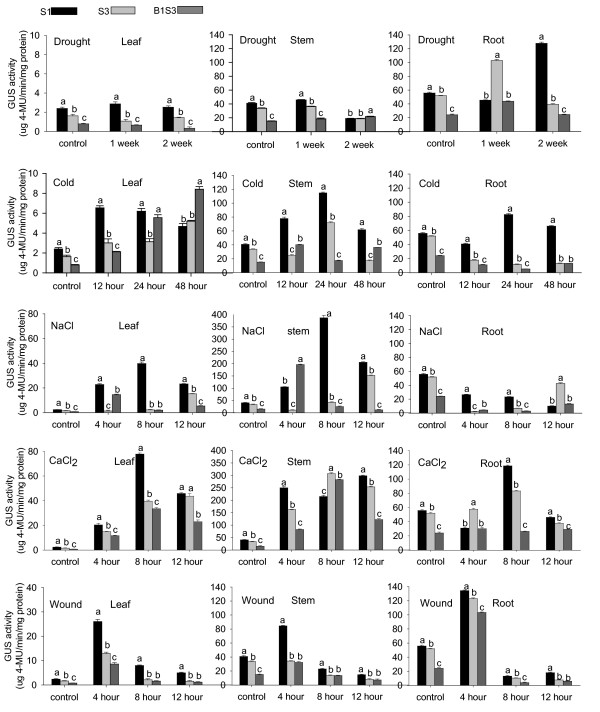
**Quantification of GUS relative activities of various *AmCBL1 *promoter segments under abiotic stresses**. Transgenic tobacco driven by promoter-GUS fusion constructs S1, S3 and B1S3 were chosen for quantification assays. Details of stress treatments are given in Table 7. The wild type tobacco without any treatment was used as the control. Leaf, stem and root samples from transgenic lines were mixed and ground with liquid nitrogen, and then utilised for further experiments. The data were measures of three independent single-copy transgenic lines, and each experiment was replicated three times. The GUS assay is described in Materials and Methods. Significance tests were performed by SPSS 16.0. Statistical analysis was performed using least significant difference and homogeneity of variance tests by SPSS 16.0, and one way ANOVA test was used for statistical analysis. Lowercase letters present the significance of differences. Error bars on the graph represent SE with three replicates.

Abiotic stresses are known to regulate *AmCBL1 *gene expression (data not shown). Moreover, database analysis predicted the existence of abiotic-stress-responsive elements. To investigate the subtle impact of abiotic stresses on gene expression of *AmCBL1 *promoter segments in tobacco, the fluorometric assay for GUS activity was also used in this experiment. There was a distinct gene expression profile for each of the three *AmCBL1 *promoter segments (Table 5). Although each of the three promoter segments responded to drought, cold, wounding, salt and CaCl_2 _treatments, respectively, there was no measurable GUS reporter enzyme increase for S1, S3 and B1S3 with salt treatment; and for S3 and B1S3 with cold treatment in roots. In addition, similarly to the full-length promoter, promoter activities of S3 and B1S3 could be greatly enhanced by application of CaCl_2_, cold, wounding or salt. However, for drought stress treatment, enhancement was not obvious despite a slight increase of GUS activities (Figure. 6).

**Table 5:** Stress induction expression profile of transgenic tobacco fragments of *AmCBL1 *promoter

stress	segment deletion analysis											
	
	S1			S3			B1S3			PBI-35S-GUS		
	leaf	stem	root	leaf	stem	root	leaf	stem	root	leaf	stem	root
Drought	+	+	+	+	+	+	+	+	+	-	-	-
Cold	+	+	+	+	+	-	+	+	-	-	-	-
Nacl	+	+	-	+	+	-	+	+	-	-	-	-
Cacl_2_	+	+	+	+	+	+	+	+	+	-	-	-
Wound	+	+	+	+	+	+	+	+	+	-	-	-

Thus, we conclude that the *AmCBL1 *promoter segment positively responded to drought, cold, wounding, salt and CaCl_2_, and that the *AmCBL1 *promoter full-length segment had the strongest induction pattern. Since the roots of deletion segments S3 and B1S3 did not respond to cold, whereas the full-length promoter did, this might result from the absence of cold-related cis-acting elements causing sequence deletion. We suggest that cold-related cis-elements may be required for response to cold in roots outside the region contained in the B1S3 construct. We also conclude that the absence of salt-related cis-elements in the *AmCBL1 *promoter, which regulate gene expression in root from the result of three promoter segment, did not respond to salt treatment in roots.

### Effects of plant hormones on gene expression of *AmCBL1 *promoter segments in tobacco seedlings

**Figure 7:**
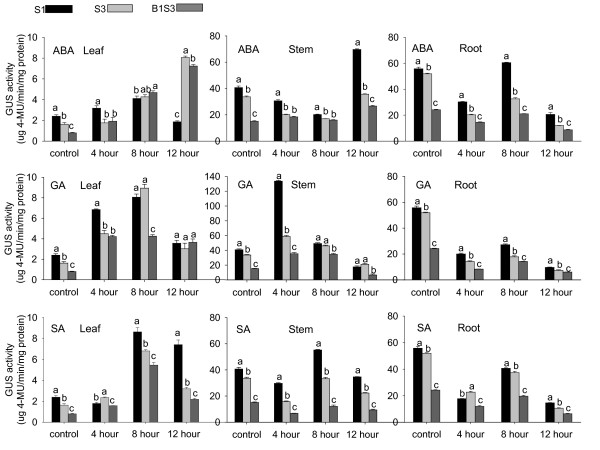
**Estimation of GUS relative activities of various *AmCBL1 *promoter segments under plant hormone induction**. Transgenic tobacco driven by promoter-GUS fusion constructs S1, S3 and B1S3 were chosen for quantification assays. The wild type tobacco without any treatment was used as the control. Details of hormone treatments are given in Table 7. Leaf, stem and root samples taken from transgenic lines were mixed and ground with liquid nitrogen, and then utilised for further experiments. The data were measures of three independent single-copy transgenic lines, and each experiment was replicated three times. The GUS assay is described in Materials and Methods. Significance tests were performed by SPSS 16.0. One way ANOVA was used for statistical analysis. Lowercase letters present the significance of differences. Error bars on the graph represent SE with three replicates.

When several major plant hormones were applied to three-week-old seedlings grown under light, distinct gene expression profiles were found for each of the three *AmCBL1 *promoter segments (Table 6). GUS expression responded differently in these treated seedlings (Figure. 7). Among the three promoter segments, only the full-length promoter S1 significantly responded to exogenous ABA induction in leaves, and also in stems and roots. It is interesting that the full-length promoter segment positively responded to ABA induction, while no positive response was detected in roots of the promoter deletion segment under ABA treatment. This implied that an ABA-related cis-acting element required for response to ABA in roots might exist outside the region of the B1S3 construct. Exposure of these transgenic seedlings to GA and SA failed to induce GUS activities in roots of the three constructs. Moreover, SA induction resulted in no increase in GUS activity in stems of transgenic plants S3 and B1S3. We suggest that the *AmCBL1 *promoter did not contain SA- and GA-related cis-elements that regulate gene expression in root, and that the deletion sequence might contain a SA-related cis-acting element that regulated gene expression in stems. In addition, the *AmCBL1 *promoter full-length segment had the strongest induction pattern compared to other promoter deletion segments.

**Table 6:** Plant hormone induction expression profile of transgenic tobacco fragments of *AmCBL1 *promoter

hormone	segment deletion analysis											
	
	S1			S3			B1S3			PBI-35S-GUS		
	
	leaf	stem	root	leaf	stem	root	leaf	stem	root	leaf	stem	root
ABA	+	+	+	+	+	-	+	+	-	-	-	-
GA	+	+	-	+	+	-	+	+	-	-	-	-
SA	+	+	-	+	-	-	+	-	-	-	-	-

## Discussion

Genes can be expressed in most plant tissues during most phases of growth and development with constitutive promoters such as CaMV35S, taking advantage of limiting temporal and spatial regulation which is suitable for proof-of-concept experiments. However, the presence of transferred genes driven by constitutive promoters may result in homology-dependent gene silencing, particularly when the promoter is also highly active [28]. Vascular-specific promoters and inducible-promoters are highly organised sequences of events that require the correct spatial and temporal expression of specific sets of genes leading to the development of a primary vascular network [29]. The unique advantages of these promoters derived from plant genes make them a potentially powerful tool for improving plant resistance to abiotic stresses, offering an interesting alternative to costly and environmentally harmful chemical control. Hence, tissue-specific and inducible promoters are preferred as experimental tools to analyse the effects of transgenic expression and produce transgenic plants with resistance to diverse abiotic stresses. However, few shuttle promoters have been described up to now. In this respect, the *AmCBL1 *promoter possesses interesting and original properties of possible practical value in biotechnological applications, especially for economically valuable dicotyledons.

A typical promoter contains a TATA-box and a CAAT-box. The function of a TATA-box is mainly the precise initiation of transcription. The CAAT-box is frequently focused on controlling transcription initiation [30]. A typical promoter also harbours some special DNA sequences; cis-acting elements inhibiting or activating gene transcription by combining with the transcription factor. In our experiment, PLACE and PLANTCARE database analysis of the promoter sequence of *AmCBL1 *and *AtCBL1 *genes showed that the *AmCBL1*, *AtCBL1 *and *AtRD29A *promoters harboured multiple-stress cis-acting elements (Table 2). It is interesting that the cis-acting elements predicted in the *AmCBL1 *and *AtCBL1 *promoters were nearly identical, despite their low homology of 40.61%. Our study of sequence analysis failed to find comparable sequences to the *AmCBL1 *promoter in other promoters from other stress-resistant desert plants; it is most likely that studies on other desert plant promoters were not adequate to allow such detection.

It has been reported the drought-induced element DRE (dehydration responsive element) usually exists upstream of these drought-induced gene promoters, while the ABA-induced gene promoter usual harbours an ABRE (ABA responsive element) [31,32]. However, other genes (e.g. *RD22A*) contain neither DRE nor ABRE elements, even if they can be induced by drought and ABA. This kind of gene expression has been reported as regulated by MYB and MYC recognition motifs [33]. *AmCBL1 *could be induced by drought, salt and ABA (data not shown), and we found some homologous sequences which could be recognised by MYB and MYC elements and one homologous sequence of the ABRE element. It is conjectured that *AmCBL1 *possibly has a specific regulatory system distinct from both *RD22A *and ABA-induced gene promoters, i.e. the transcription factors MYB, MYC and ABRE rather than DREB played a key role in upstream expression regulation of the *AmCBL1 *promoter.

Sequence BLASTing of the *AmCBL1 *promoter and the 5'-untranslated region of *AmCBL1 *cDNA showed that a 690-bp intron existed upstream of the 5'-end, 63 bp away from the initiator ATG of the *AmCBL1 *gene. BLASTing the 5'-flanking region of *AtCBL1 *with its 5'-untranslated region identified a 411-bp intron upstream of the 5'-end, 123 bp away from the initiator, which was similar to the result for *A. mongolicus*. Introns have also been discovered on genes *AtCBL1-4 *and *AtCBL9 *in the *Arabidopsis CBL *gene-family [15]. It is interesting that introns were found on the 5'-end of both the *AmCBL1 *and *AtCBL1 *promoters. Genome-wide analysis in *Arabidopsis thaliana *revealed that the degree to which an individual intron matches the promoter-proximal intron profile is a strong predictor of its ability to increase expression. Sequences responsible for elevating expression are dispersed throughout an enhancing intron [34]. In many cases, introns have a larger influence than promoters in determining the level and pattern of expression [35-38]. Function of this 690-bp intron, i.e. whether it plays a role as an enhancer or if it alters tissue-specific expression, is of interest to us.

In this study, we performed GUS-assay experiments and detected sharp decreases in leaf, stem and roots by the deletion of the 5'-UTR intron, compared with GUS activities detected in S3. However, deletion by removing 611-bp from the 5'-end of the full-length *AmCBL1 *promoter under normal conditions without any stress induction, was comparable to reported intron-mediated transcription enhancement in transgenic rice cells [39] and mammal cells [40]. Previous studies in plants failed to detect the requirement of introns for the establishment of a C4-specific expression pattern of the *ppcA1 *gene[41], most likely because the regulatory mechanism between desert plants and crops differ. In addition, no stain was detected when 129 bp was deleted from the B1S3 3'-end, indicating that some positive-regulatory element might exist there. Overall, our results experimentally demonstrated for the first time that there were cis-acting elements located in the 611-bp promoter deletion sequence and that an enhancer-like sequence existed in the proximal intron in a desert plant.

Razorblade sections indicated that the *AmCBL1 *promoter was a phloem-specific promoter. GUS activity of *AmCBL1 *promoter segments were strictly localised in phloem of the promoter deletion segments of transformed tobacco with differing staining strengths. It is interesting that GUS activity did not occur in xylem but only in phloem, so long as the cambium was formed. Moreover, root sections showed that staining was not only in the vascular bundle but also in the root tip meristematic zone. It was also interesting that GUS staining did not occur in root cap, which differed to results for the CaMV35S promoter. The mechanism behind this phenomenon is still unclear. It is predicted that this tissue-specific alteration contributed to evolution of desert plants. Hehn identified a highly conserved motif 'ATAAGAACGAATC' involved in the phloem strength and specificity was [42]. Other vascular-specific promoters from rice, Milk Vetch Dwarf Virus [43] and pumpkin PP2 gene promoter [44] also contained this conserved sequence. In all these promoters, this motif was upstream of the TATA box [45]. Consistent with this notion, a similar motif (298/310) was also found 262 bp upstream from the TATA box in the *AmCBL1 *promoter. Consequently, this confirmed that the motif 'ATAAGAACGAATC' and its homologue sequences determined the promoters' phloem-specific expression pattern.

Cis-acting regulatory elements are important molecular switches involved in the transcriptional regulation of dynamic networks of gene activities controlling various biological processes, including abiotic stress responses, hormone responses and developmental processes. The ever-improving accuracy of transcriptome expression-profiling has led to the identification of various combinations of cis-acting elements in the promoter regions of stress-inducible genes involved in stress and hormone responses [45]. Here, we discuss major cis-acting elements that are vital parts of gene expression in stress and hormone responses elements by fluorometric analysis of GUS activity of *AmCBL1 *promoter segments. The *AmCBL1 *promoter segment positively responded to drought, cold, wounding, salt, CaCl_2_, ABA, GA and SA. Moreover, the *AmCBL1 *promoter did not contain salt-, SA- and GA-related cis-elements which regulated gene expression in roots. It was also revealed that cold- and ABA-related cis-elements in roots and SA-related cis-elements in stems might exist outside the region contained in the B1S3 construct. Ma confirmed stress-related 5'-cis-elements on a genome-wide scale, and placed the stress response within the context of tissues and cell lineages in the *Arabidopsis *root [46]; evolutionary pressures may have conferred distinct responses to different stresses in time and space. In this regard, our induction promoter *AmCBL1 *has advantages over *AtCBL1 *for molecular breeding of economically valuable to select superior plants for harsh environments.

## Conclusions

In the present study we clarified that the *CBL1 *gene promoter from *A. mongolicus *is a vascular-specific promoter (particularly phloem-specific) by histochemical assay and razorblade section. Further analysis of GUS activity showed that the *AmCBL1 *promoter could be induced by multiple abiotic stresses (dehydration, cold, salinity, CaCl_2 _and wounding) as well as by plant hormones (ABA, GA and SA). Drought and cold tolerance are especially advantageous to plant growth in harsh environments.

Deletion mapping of the 5'-end and site-specific mutagenesis identified four regions of the promoter essential for expression under the five stress conditions and in response to three plant hormones. Some sequence elements were important for response to all stress treatments, whereas others were stress-specific. We demonstrated that the B1S3 fragment was sufficient to confer the stress induction and tissue-specific developmental expression characteristics of the *CBL1 *gene promoter to a GUS reporter gene.

Fluorometric analysis of GUS activity of the *AmCBL1 *promoter segments (without any stress or hormone treatment) showed that the 611 and 690-bp introns and 129-bp sequence outside the B1S3 region harboured cis-acting elements. This resulted in significant decrease in expression level in transgenic tobacco, and the most critical region essential for expression of the *AmCBL1 *promoter under all five environmental stresses and for the three plant hormones was located within the 5'-UTR intron. It was further demonstrated that the intron on the 5'-UTR played a key role in determining the promoter expression strength, which clearly indicated that this intron was involved in regulating expression levels. Moreover, fluorometric analysis of GUS activity of the *AmCBL1 *promoter segments under five stress and three hormone treatments revealed that the *AmCBL1 *promoter did not contain salt-, SA- and GA-related cis-elements, which regulated gene expression in roots. It was also revealed that cold- and ABA-related cis-elements in roots, and SA-related cis-elements in stems, might exist outside the region contained in the B1S3 construct.

Further studies will be necessary to elucidate the mechanism behind the sharp decrease in expression activity caused by the 5'-UTR intron deletion. That is to say, that both a deletion construct only covering the untranslated region of the promoter and a deletion construct without the UTR should be included in further GUS analysis. Despite the poor understanding of action mechanisms, further studies will hasten transformation of economically important plants with the *AmCBL1 *promoter construct fused with the calcium sensor *CBL1*, which differentially regulates drought, salt and cold responses.

## Methods

### Plant material

*A. mongolicus *seeds were collected from Alashan, Inner Mongolia Autonomous Region, China. Healthy seeds of *A. mongolicus *were surface sterilised with 70% ethanol for 30 s and then with 10% sodium hypochlorite for 20 min. The seeds were rinsed thoroughly with distilled water, and then placed in 150 mL pots containing 50 mL of MS medium and 0.7% (w/v) agar, pH 5.8, which was autoclaved at 121°C for 20 min. The seeds were incubated at 25°C with a 16/8 h light/dark photoperiod for 14 d, by which time their two cotyledons were completely unfolded. Two-week-old cotyledons were used for promoter cloning. Desiccation stress was simulated by drying the seedlings on filter paper for 4 h. Then cotyledons were frozen in liquid nitrogen and kept at -80°C.

### Amplification of *AmCBL1 *promoter

Genomic DNA was extracted from the Cotyledons of *A. mongolicus*, and used as templates for anchored PCR (A-PCR) amplification. The 5'-flanking region of the *AmCBL1 *gene was isolated using a new method for chromosome walking A-PCR [47]. Amplification procedures are described in Table 1. Sequences of DNA adaptors and primers used for promoter amplification are shown in Table 2.

### Construction of chimeric promoter

Deletions were made at the 5'-upstream end, based on the distribution of structural and expressional elements of the known *AmCBL1 *promoter sequence. The detailed profiles of vector construction are shown in Figure. 2. To construct the various length deletions of *AmCBL1 *promoter/GUS fusion products, a PCR series was carried out with six pairs of primers, HS1/RS, HS2/RS, HS3/RS, S3/Bin1 and S3/Bin2 (Table 2), respectively. Five different length promoters were released by HindIII and SamI digestion. Then the CaMV35S promoter of PBI121 was separately replaced by the above released fragments. Five expression vectors containing various lengths of *AmCBL1 *promoters were individually obtained and designated S1, S2, S3, B1S3 and B2S3. In addition, the CaMV35S promoter was the positive control and wild-type tobacco the negative control, in order to determine *AmCBL1 *promoter activity. DNA manipulation and cloning were performed [48].

### Transient expression

The expression vector constructs S1, S2, S3, B1S3, B2S3 and PBI-35S were introduced into *Agrobacterium tumefaciens *strain EHA105 by freeze-thaw method. Leaf discs of *Nicotiana tabacum *L. cv. 89 and cotyledons of *A. mongolicus *were infected using *Agrobacterium tumefaciens *containing various segments of *AmCBL1 *promoter and cultured at 25°C in darkness for 4 d. They were then transferred into X-Gluc at 37°C overnight, and the chlorophyll removed from green tissue by incubating in 75% ethanol.

### Tobacco transformation and PCR analysis

PBI121 plasmids containing the promoter-GUS fusion constructs were transferred from *Escherichia coli *PMD18-T into *Agrobacterium tumefaciens *strain EHA105 for transformation of tobacco. Transformation of *N. tabacum *L. cv. 89 was performed [49]. Primary transgenic explants were grown in the tissue culture chamber at 25°C under a 16/8 h light/dark cycle. The transgenic plants were screened for integration of the intact promoter-GUS chimeric gene into the genome DNA by PCR. PCR products were analysed on 1% (w/v) agarose gel.

Total genome DNA was isolated from leaves of kanamycin-resistant tobacco plants using the Tiangen plant DNeasy kit and was used as a template. PCR analysis was carried out using the primer pair: GUSS:: GTCACTCATTACGGCAAAGT/GUSR:: CAGCAGCAGTTTCATCAATC. Transgenic copy number was assayed by relative quantification real time PCR. SYBR Green was used in this test [50]. NRA was used as the reference gene. Primer pairs: NRAF:: TCTTGAAAGATCACCCCGG/NRAR:: CCAGGAGAGTCAGAGGTGTA and GUSF:: GCTGTGCCTGAACCGTTATTA/GUSRR:: CACTGATACTCTTCACTCCAC were used in real time PCR.

### Histochemical GUS staining

GUS histochemical staining of the three-week-old transgenic tobacco plants containing *AmCBL1*::GUS fusion constructs followed a previously described method [51]. The images of blue-coloured whole plants were pictured by a Canon scanner. The GUS-positive plant tissues were examined with a light microscope (Leica) at a low magnification and photographed with a digital camera. In addition, roots, leaf sheaths and stalks were sectioned manually with a razorblade and the sections stained with X-Gluc as described [52]. GUS-stained tissues and plants in the present paper represent the typical results of at least three independent transgenic lines for each construct.

### Stress and hormones induction

Tissue samples in all stress experiments described were taken after different induction times, immediately frozen in liquid nitrogen, and stored at -80°C until use. For each stress induction of transgenic promoter segments (Table 7), at least five plants of three-week-old PCR-positive tobacco were selected. For drought treatment, the tobacco plants were planted in sand, supplemented with 0, 1 or 2 weeks of drought at room temperature under a growth regime of 16/8 h light/dark. Similarly, the low-temperature stress seedlings were incubated in 16/8 h light/dark for 8, 24 or 48 h at 4°C, respectively. For the wounding treatments, stems, leaves and roots of transgenic tobacco were cut into pieces, and then cultured on 1/2 MS liquid medium for 4, 8 or 12 h, separately. The high-salt, CaCl_2_, ABA, GA and SA treatments were achieved by moving the seedlings to 1/2 MS liquid medium for 12 h, and then exposed to 1/2 MS medium either containing 200 μmol/L NaCl, CaCl_2_, ABA, GA or SA; these stress treatments continued for 4, 8 or 12 h, respectively. Seedlings without any induction were used as controls. All the above treatments were carried out under a growth regime of 16/8 h light/dark at 20 ± 1°C unless otherwise mentioned.

**Table 7:** stress treatments of transgenic tobaccos directed by construct S1, S3, B1S3

Stress	Time	Deletion analysis			
		
		PBI-35S-GUS	S1	S3	B1S3
Drought(water deficiency)	0,1,2 week	▲	▲	▲	▲
Cold(4°C)	0,12,24,48 h	▲	▲	▲	▲
ABA(200 μmol/)	0,4,8,12 h	▲	▲	▲	▲
GA(200 μmol/)	0,4,8,12 h	▲	▲	▲	▲
SA(200 μmol/)	0,4,8,12 h	▲	▲	▲	▲
Nacl(200 μmol/L)	0,4,8,12 h	▲	▲	▲	▲
Cacl2(200 μmol/)	0,4,8,12 h	▲	▲	▲	▲
Wound	0,4,8,12 h	▲	▲	▲	▲

### Protein extraction and fluorometric GUS assays

Assays for GUS activity in transgenic tobacco leaves with main vein, stems and roots were performed based on the protocol of Jefferson [53] using 4-methyl-umbelliferyl-glucuronide (Sigma) as a substrate for the fluorescent assay. The protein concentration of the extracts were determined utilising BSA as a standard protein as described by Bradford [54]. Fluorescence was measured in a microplate spectrofluorometer (Megellan, Taken). The excitation wavelength was 365 nm and the emission wavelength 455 nm. Each assay was repeated three times. The data presented were collected from at least four independent lines for each construct. Since 4-methylumbelliferone solution decays rapidly during storage, the 4-MU stock solution was only used for assays 2-3 times.

### Computer analysis

DNA sequences were analysed by software DNAMAN, and the promoter elements were analysed by PLACE http://www.dna.affrc.go.jp/PLACE/ and PLANTCARE http://bioinformatics.psb.ugent.be/webtools/plantcare/html/. Sequence alignments were created with the program DIALIGN. Differences in GUS activity among treatment groups were tested with analyse of variance (ANOVA) in SPSS 16.0

## Authors' contributions

YY carried out the promoter cloning, sequence alignment and vector construction. LG carried out the tobacco transformation, histochemical and quantitative GUS assays, and drafted the manuscript. XX and WY conceived the study, participated in its design and coordination, and helped draft the manuscript. All authors read and approved the final manuscript.

## Supplementary Material

Additional file 1 ***AmCBL1 *cDNA sequence**. The blue sequence represents 5' UTR of *AmCBL1 *gene. Click here for file

Additional file 2 **BLAST result**. Blast result of *AmCBL *5' flanking region and *AmCBL1 *5'UTR. Symbol "*" written between lines name to identify the same sequence. Click here for file

Additional file 3 **transient expression**. Transient expression of series *AmCBL1 *promoter deletion segments of *A. mongolicus *and tobacco. Transient GUS expression of various *AmCBL1 *promoter constructions: S1, S2, S3, B1S3 and B2S3. 35S was the positive control. Am represents *A. mongolicus *and To represents tobacco. The GUS staining method is described in Materials and Methods. This experiment was repeated at least three times. Click here for file

Additional file 4 **PCR**. PCR identification of GUS fusion using kanamycin-resistant plants. M: DL2000 marker, CK1: positive control, CK2: wild-type tobacco, S11-S15: transgenic tobacco of S1-GUS; S21-S25: transgenic tobacco of S2-GUS; S31-S35: transgenic tobacco of S3-GUS; B11-B15: transgenic tobacco of B1S3-GUS; B21-B25: transgenic tobacco of B2S3-GUS; G1-G5: transgenic tobacco of CAMV35S-GUS. These PCR products were amplified with pairs of primers: GUSS/GUSR. Click here for file

Additional file 5 **qPCR**. Real-time relative quantitative PCR testing the copy number of transgenic plants. Transgenic copy number between 0.5 and 1.5 were chosen for further analysis as single-copy transgenic lines. S1-1, S1-5 and S1-6: single-copy transgenic lines of S1-GUS; S3-1, S3-2 and S3-4: single-copy transgenic lines of S3-GUS; B1S3-1, B1S3-2 and B1S3-3: single-copy transgenic lines of B1S3-GUS; 35S-1, 35S-4 and 35S-5: single-copy transgenic lines of CAMV35S-GUS. PCR products were amplified with pairs of primers: GUSF/GUSRR. SYBR Green was used in this test, and NRA was used as the reference gene. Error bars on the graph represent SE. There were three replicates. Click here for file
