# Supplementary material for: Identification and functional characterisation of the promoter of the calcium sensor gene CBL1 from the xerophyte Ammopiptanthus mongolicus
Source: BMC Plant Biol. 2010 Jan 29;10:18. doi: 10.1186/1471-2229-10-18 (PMC2844064; doi:10.1186/1471-2229-10-18)
Supplement: Additional file 1 — AmCBL1 cDNA sequence. The blue sequence represents 5' UTR of AmCBL1 gene. [file 1471-2229-10-18-S1.PDF]

| | | | | | | |
|---|---|---|---|---|---|---|
| 1 | TATAGCCCAA | CTCACTCAAA | TGCCATTTTT | CTAGGACAAA | GTTCTTTACT | TTTTCAACTG |
| 61 | GTGAAGTGGT | GAAGCCTGAG | CACTGCTCCG | GTACCTTGCT | GCTTTGCCTG | GATTTCCTCC |
| 121 | ATAAATGGGC | TGCTTCAACT | CTAAGGTTAG | GAGACAGTTT | CCTGGGCAGG | AGGATCCAGT |
| 181 | AATTCTTGCA | TCACAGACAG | CTTTCACTGT | CAGCGAGGTT | GAAGCACTAT | TTGAGCTTTT |
| 241 | TAAGAGCATC | AGCAGTTCTG | TCATAGATGA | TGGACTAATA | AGCAAGGAAG | AATTTCAATT |
| 301 | GGCAATTTTC | AAAAATAGGA | AAAAAGAAAA | TATCTTTGCA | AATCGGATCT | TTGATCTATT |
| 361 | TGATGTTAAG | AAGAAAGGAG | TCATTGATTT | TGACGACTTT | GTTAGATCAC | TCAACGTCTT |
| 421 | CCACCCAAAT | GCACCACTAG | AAGACAAGAT | AGATTTTTCT | TTTAAGCTTT | ATGATTTGGA |
| 481 | CAATACAGGA | TTTATAGAGC | GCCAAGAGGT | CAAGCAAATG | TTAATTGCGC | TTCTTTGTGA |
| 541 | GTCTGAAATG | AAGTTGGCTG | ATGAGGTGAT | AGAAACAATT | CTTGACAAGA | CTTTCTTGGA |
| 601 | TGCTGACCTG | AACCAAGATG | GGAAAATAGA | CATTGTCGAG | TGGCAAAATT | TTGTTTCTAA |
| 661 | AAATCCATCA | CTGCTTAAAA | TCATGACCCT | ACCCTATCTG | AGGGACATAA | CAACTTCTTT |
| 721 | CCCAAGTTTT | GTATTTAACT | CTAATGTGGA | TGAAGTTGCT | GCTTGAGCTC | ACGGCTTTAC |
| 781 | GTTATGCAAT | TAATGAGAAA | ATTGGGTGAG | TGTAATATAT | AAGAGCTTAA | AGAGTGATGA |
| 841 | AGCAATAGAT | GCTTGATGAA | GTTAAGTTTT | TTTTTCTTTC | TACAATGGTG | GCAATGAGGT |
| 901 | TTGAACTT | | | | | |
