# Supplementary material for: Identification and functional characterisation of the promoter of the calcium sensor gene CBL1 from the xerophyte Ammopiptanthus mongolicus
Source: BMC Plant Biol. 2010 Jan 29;10:18. doi: 10.1186/1471-2229-10-18 (PMC2844064; doi:10.1186/1471-2229-10-18)
Supplement: Additional file 2 — BLAST result. Blast result of AmCBL 5' flanking region and AmCBL1 5'UTR. Symbol "*" written between lines name to identify the same sequence. [file 1471-2229-10-18-S2.PDF]

```
1     TTATTTATTTTAAAATATTTATTTTAAAATGGATTTAATTGGTATAAATTATTAGTGTAA
1     ............................................................
61    AAAATTTTTTATACTGTCAATCAATCATAAATTATCACATTAATTAAAATGTTTGATTCT
1     ............................................................
121   TATGCTAACTATTTTAAAAATCACCCAAATCATGAATATATATTATATTTATATTTGACA
1     ............................................................
181   ACATTGTACATAATATTATATTCAAATTTACTCATTTTCAACTTCATTGTTATTATAAAA
1     ............................................................
241   AAAACTTCATTGTTATTGAGCTATAACTAAAGGAGAATGCTAATTAGTATCCTTTGAACA
1     ............................................................
301   TTGGTTAAGAAAACAAGAAAAAAAAATTTTGTATTGAAAGATATATATTTAATATTTTAAA
1     ............................................................
361   AAATGTAACACTTAATTTTTTAGGGAAACATTTCTTTTTATTGATTTATTAATCAATATT
1     ............................................................
421   CTTAATGAGATCAGTTACTAAATTAGAGCTGATGTAAATAAATAAATAATTATCAATAAG
1     ............................................................
481   AACGATATATTTTTTATTATTGAATGAATTTTAGAGGTAACTACTCTCTTAATAAGTGGG
1     ............................................................
541   TCTCAAGAATCTGTTTAACGAGTTAATAAAGAGTGGGGACCCGTTTTCTACGGCCACCTT
1     ............................................................
601   CACCAAACCAAACACAATTCGCCGTTAACGTTTCAAACTTTACTTTCAGTTCAGGAACTT
1     ............................................................
661   TCTGACAACAACAGCGTGAGTCTAATCTCTCTCTCTTTCACCCTGATGTACTATGTGTGT
1     ............................................................
721   ATGTATATATAAGAGAGAGAGAGAGAGGGATGGTTCTAAATAAATAAAACTCATACCTCAGT
1     ............................................................
781   TCTACTCTCTCAGCTCAACCCACTTTCCCTTTTCTCGAGTACTCCGCGTTTTCTTTGTTT
1     ............................................................

841   TTCTCTCTCCTCTTTTTTCTCTCATTTCTTATAGCCCAACTCACTCAAATGCCATTTTTC
                                   *******************************
1     .............................TATAGCCCAACTCACTCAAATGCCATTTTTC

901   TAGGACAAAGTTCTTTACTTTTTCAACTGGGTGAGTCTTTTTCCATCATCACCGTTCTTT
      *****************************
32    TAGGACAAAGTTCTTTACTTTTTCAACTGG..............................

961   TTTCGTTTGATTTACATCTTATGTTGAGGTTCTTTTCCGTGGGGTTTATGGGAGGAGGGG
62    ............................................................
1021  TAGAAAAAAAATAATAGAATGTGGGTTTCATTTTTTTCTGATTAATTATGTGATGTTTAT
62    ............................................................
1081  GTATTTTTTTTGTTACACCGATCTTCTTGACGTCTTTTGATTTCTAGATGATGTTTATAA
62    ............................................................
1141  AGTTATGAATTTGCGCTTCTTTTGATAGGATCCATGGATTTCATCTTGGTATTGGTTGGT
62    ............................................................
1201  CTTTAATTTTTGTAAGATTATTATTATTATTATTATTTCATCTTGGTAATGGGGTAAAGA
62    ............................................................
1261  AATTTTAGTCAAGATCAAATTTTTATTTATTAATCACTCAATTTGTTTATTATTTGTTTT
62    ............................................................
1321  CTTGAATTATTATGTAAGTTGTGTCTTTTTGTGTCCCTAAGCTCATGGTCATAGCCATCT
62    ............................................................
1381  TTTCATAATGATCAATTATTGTTCTGAGTCACATACAGTGTTGGAACACTACATACTACA
62    ............................................................
1441  GTTATTCTTTTTTAAGATTCTTCTGCTTTATCTTTTATTTTTTTTAATTCAATGTTTAGC
62    ............................................................
1501  ACAGCTAACATGTACCAAAACTTCACAAGGTTGATTTGTTTATTTATTATTTTGCGTTGT
62    ............................................................
1561  TGGTGTTTCAATTTGGGATTAAAATTGCAAGGTTAAGATGATTTTGGTGATATATTTTAG
62    ............................................................

1621  TGAAGTGGTGAAGCCTGAGCACTGCTCCGGTACCTTGCTGCTTTGCCTGGATTTCCTCCA
      ************************************************************
62    TGAAGTGGTGAAGCCTGAGCACTGCTCCGGTACCTTGCTGCTTTGCCTGGATTTCCTCCA

1681  TAAATGGGGTGCTTCAACTCTAAGGTTAGGAGACAGTTTCCTGGGCAGGAGGATCCAGTA
      ***
122   TAA.........................................................

1741  ATTCTTGCATCACAGACAGCTT
```
