# Supplementary figures and images for: Identification and functional characterisation of the promoter of the calcium sensor gene CBL1 from the xerophyte Ammopiptanthus mongolicus
Source: BMC Plant Biol. 2010 Jan 29;10:18. doi: 10.1186/1471-2229-10-18 (PMC2844064; doi:10.1186/1471-2229-10-18)

S1

S2

S3

B1S3

B2S3

35S

Am

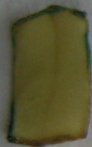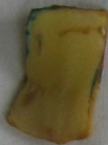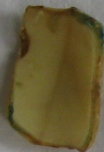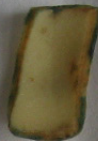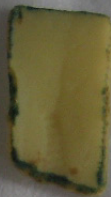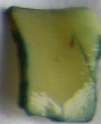

To

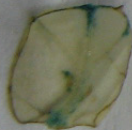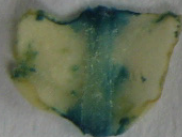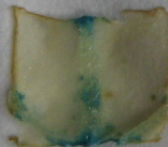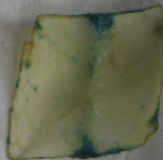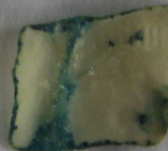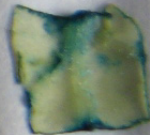

Supplement: Additional file 3 — transient expression. Transient expression of series AmCBL1 promoter deletion segments of A. mongolicus and tobacco. Transient GUS expression of various AmCBL1 promoter constructions: S1, S2, S3, B1S3 and B2S3. 35S was the positive control. Am represents A. mongolicus and To represents tobacco. The GUS staining method is described in Materials and Methods. This experiment was repeated at least three times. [file 1471-2229-10-18-S3.PDF]

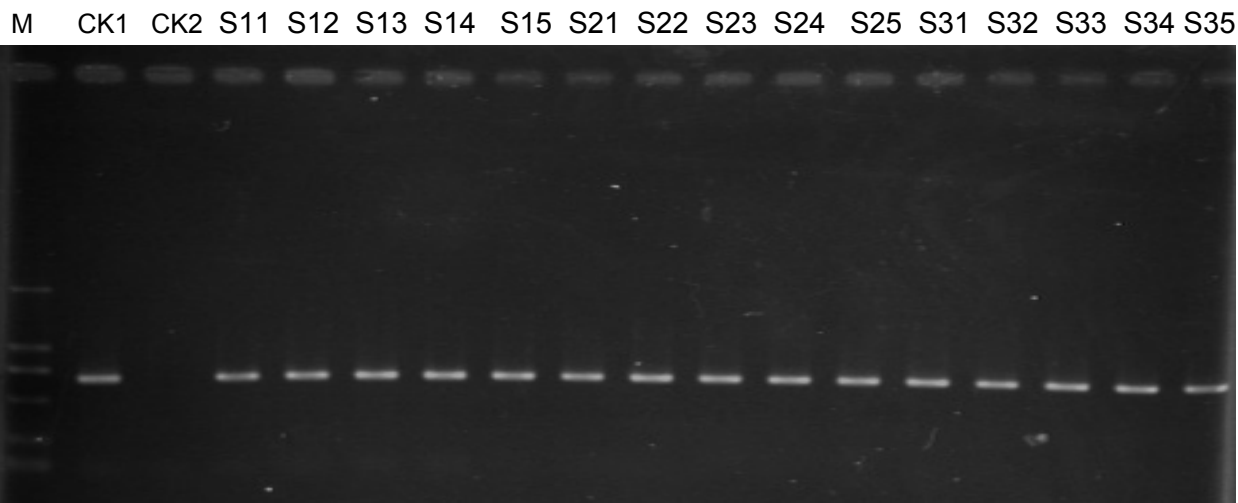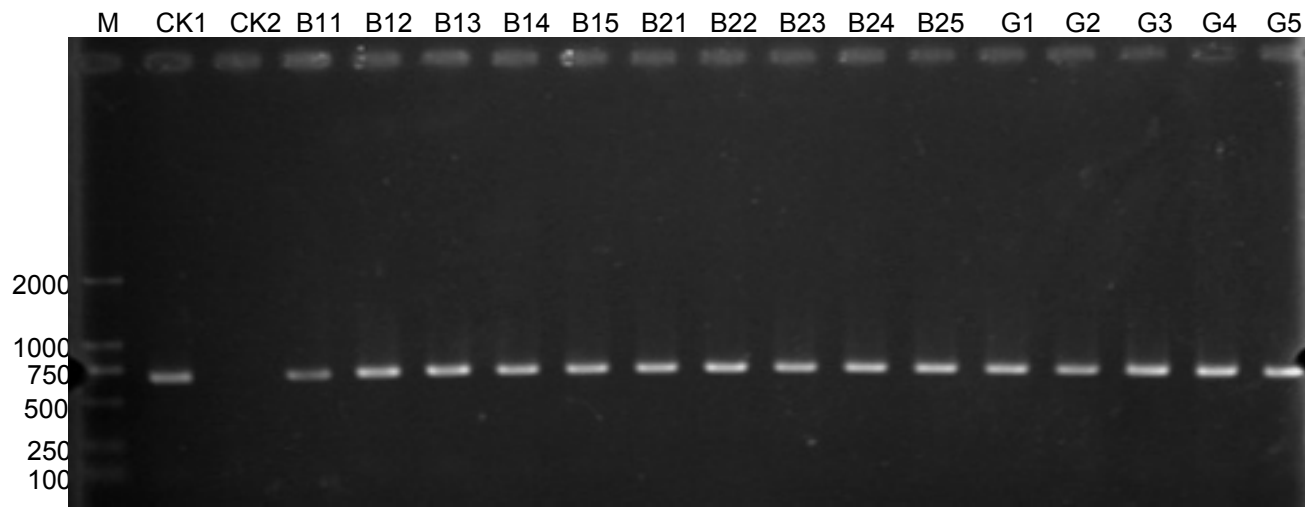

Supplement: Additional file 4 — PCR. PCR identification of GUS fusion using kanamycin-resistant plants. M: DL2000 marker, CK1: positive control, CK2: wild-type tobacco, S11-S15: transgenic tobacco of S1-GUS; S21-S25: transgenic tobacco of S2-GUS; S31-S35: transgenic tobacco of S3-GUS; B11-B15: transgenic tobacco of B1S3-GUS; B21-B25: transgenic tobacco of B2S3-GUS; G1-G5: transgenic tobacco of CAMV35S-GUS. These PCR products were amplified with pairs of primers: GUSS/GUSR. [file 1471-2229-10-18-S4.PDF]

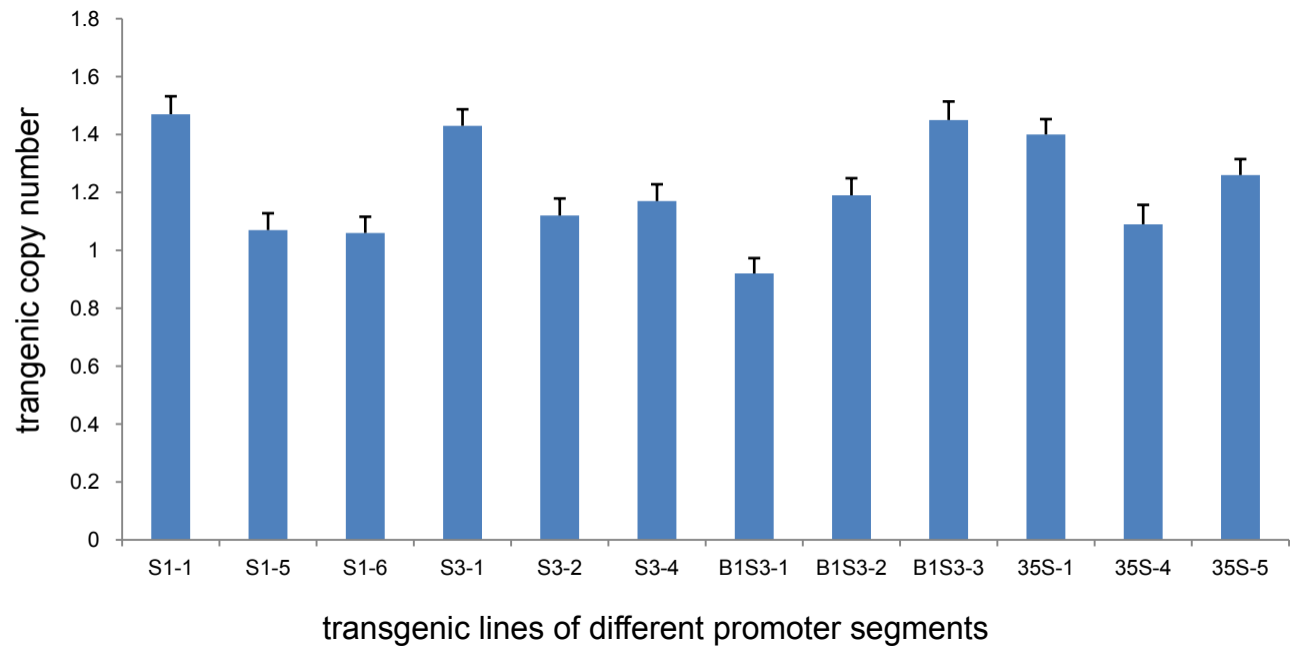

Supplement: Additional file 5 — qPCR. Real-time relative quantitative PCR testing the copy number of transgenic plants. Transgenic copy number between 0.5 and 1.5 were chosen for further analysis as single-copy transgenic lines. S1-1, S1-5 and S1-6: single-copy transgenic lines of S1-GUS; S3-1, S3-2 and S3-4: single-copy transgenic lines of S3-GUS; B1S3-1, B1S3-2 and B1S3-3: single-copy transgenic lines of B1S3-GUS; 35S-1, 35S-4 and 35S-5: single-copy transgenic lines of CAMV35S-GUS. PCR products were amplified with pairs of primers: GUSF/GUSRR. SYBR Green was used in this test, and NRA was used as the reference gene. Error bars on the graph represent SE. There were three replicates. [file 1471-2229-10-18-S5.PDF]
